# Mid to long-term effect of balloon pulmonary valvuloplasty on right ventricle function: a three-dimensional echocardiographic study

**DOI:** 10.1186/s43044-026-00798-5

**Published:** 2026-11-09

**Authors:** Ayah Tarek Elsayegh, Azza Abdallah Elfiky, Ehab Fahmy Abdelkader, Heba Mohamed Kamel

**Affiliations:** Cardiology Departement, Faculty of Medicine, Ain Shams University, Cairo, Egypt

**Keywords:** Balloon pulmonary valvuloplasty, Pulmonary valve stenosis, Right ventricular ejection fraction, Three-dimensional echocardiography, Pediatric cardiology

## Abstract

**Background:**

Right ventricular (RV) remodelling may persist after balloon pulmonary valvuloplasty (BPV) for isolated valvular pulmonary stenosis (PVS). Three-dimensional echocardiography (3DE) enables global RV volumetric assessment beyond conventional 2D indices.

**Methods:**

This cross-sectional observational study included 200 pediatric patients (≤ 18 years) with isolated PVS who underwent successful BPV (post-procedural peak gradient < 36 mmHg) at least 1 year before evaluation. All patients had clinical assessment, ECG, standard 2D/Doppler echocardiography (TAPSE, S′, FAC), and ECG-gated 3DE RV quantification to derive RV end-diastolic and end-systolic volume indices and right ventricular ejection fraction (RVEF). Patients were classified into 2 groups; group 1 had normal RVEF (≥ 45%) and group 2 had impaired RVEF (< 45%).

**Results:**

RVEF was normal in 71% (*n* = 142) and impaired in 29% (*n* = 58). Group 2 was associated with; older age at assessment (median 120 vs. 72 months, *p* < 0.001), later intervention (median age at BPV 24 vs. 3 months, *p* < 0.001), and longer time since BPV (96 vs. 68 months, *p* = 0.004). it also showed association with higher pre-dilatation peak gradients (97.24 ± 26.47 vs. 83.8 ± 24.61 mmHg, *p* = 0.017) and higher post-dilatation gradients (30.97 ± 9.81 vs. 23.56 ± 8.59 mmHg, *p* < 0.001). Group 2 also had lower conventional RV function indices: S′ 12.64 ± 3.22 vs. 15.01 ± 1.09 cm/s, TAPSE 16.28 ± 2.75 vs. 19.21 ± 1.72 mm, and FAC 39.86 ± 5.21 vs. 49.8 ± 2.62% (all *p* < 0.001). by using 3DE interrogation, group 2 showed higher RVEDV index (62.0 ± 11.06 vs. 43.14 ± 11.44, *p* < 0.001) and RVESV index (34.41 ± 8.24 vs. 22.76 ± 6.65, *p* < 0.001) .

**Conclusions:**

At ≥ 1 year after successful BPV, nearly one-third of children showed impaired 3DE-derived RVEF, associated with older age at intervention, higher residual gradients, and RV dilatation, patients with RV impairment were underestimated when assessed by 2D vs. 3D echo, supporting the role of 3DE in long-term surveillance.

## Introduction

Congenital pulmonary valve stenosis (PVS) subjects the right ventricle (RV) to chronic pressure overload, promoting hypertrophy and functional adaptation that may persist even after relief of the obstruction. Percutaneous balloon pulmonary valvuloplasty (BPV), introduced in the early 1980s, is widely accepted as the preferred intervention for isolated valvular PVS across age groups because it effectively reduces transvalvular gradients with low procedural morbidity [1]. While acute hemodynamic success is well documented, fewer studies have focused on how RV systolic performance evolves in the longer term, and which factors are associated with incomplete functional recovery despite an apparently satisfactory residual gradient [2, 3].

Echocardiography is crucial for follow-up after BPV, but right ventricular systolic function is difficult to characterize with conventional two-dimensional (2D) approaches having complex geometry and contraction patterns. Common indices—tricuspid annular plane systolic excursion (TAPSE), tissue Doppler–derived tricuspid annular systolic velocity (S′), and fractional area change (FAC)—are recommended for routine assessment, yet they reflect selected components of RV motion and can be influenced by image alignment and loading conditions [4]. These limitations are particularly relevant in pediatric cohorts and in late post-intervention assessment, where subtle residual dysfunction may be clinically important but not consistently captured by single-plane or single-parameter measurements [5].

Three-dimensional echocardiography (3DE) offers a practical method for global RV functional assessment by quantifying RV volumes throughout the cardiac cycle and deriving right ventricular ejection fraction (RVEF) without geometric assumptions [6]. Cardiac magnetic resonance (CMR) remains a reference technique for RV evaluation, but its use for serial follow-up can be constrained by availability, cost, scan time, and patient cooperation—factors that often matter in children—making 3DE an attractive tool for long-term surveillance [7].

Accordingly, this study aimed to evaluate long-term RV systolic function after successful BPV for isolated valvular PVS using 3DE, and to identify clinical and echocardiographic factors associated with RV functional outcomes at least one year after the procedure.

## Patients and methods

### Study design and setting

This cross-sectional observational study was carried out on 200 pediatric patients (≤ 18 years) with isolated valvular pulmonary stenosis (PVS) who had undergone successful BPV at least one year before evaluation. The study was conducted in the Echocardiography Laboratory, Cardiology Department, Ain Shams University Hospitals over a period of 2 years from January 2023 to January 2025.

BPV was considered successful when the pressure gradient across the pulmonary valve was reduced to < 36 mmHg [8].

### Exclusion criteria

We excluded patients with associated congenital cardiac anomalies, including hemodynamically significant shunts such as large atrial septal defect or ventricular septal defect, significant subvalvular or supravalvular pulmonary stenosis, evidence of peripheral pulmonary stenosis or peripheral pulmonary arterial tree hypoplasia, significant pulmonary regurgitation, hemodynamic instability, relevant comorbidities such as renal impairment, insufficient ultrasound image quality (defined as more than three myocardial segments not optimally visualized on conventional two-dimensional echocardiography), or refusal to participate.

### Clinical assessment and electrocardiography

All participants underwent comprehensive history taking, including demographic data, general medical history and comorbidities, prior interventions, and family history of congenital anomalies. A detailed clinical examination was performed, documenting heart rate, blood pressure, and respiratory rate, and assessing the neck veins, head and neck, extremities, chest, and abdomen. A standard 12-lead electrocardiogram was obtained at the time of echocardiographic examination to identify findings consistent with right ventricular overload, including right axis deviation and patterns of right ventricular hypertrophy [9].

### Two-dimensional and Doppler echocardiography protocol

ECG-gated transthoracic echocardiography was performed using a Vivid E95 system with M5s/6S probes (up to 4.5 MHz), selected according to patient size and age. Agitated infants and children received oral chloral hydrate (50 mg/kg) 15 min before imaging. A sequential segmental approach evaluated cardiac anatomy, associated anomalies, RV function and RV free wall thickness [10]. RV systolic function was assessed using fractional area change (FAC) using a cut value of 40% [5], as well as tricuspid annular plane systolic excursion (TAPSE) by M-mode and tissue Doppler–derived tricuspid annular systolic velocity (S′), with S′ <10 cm/s indicating systolic dysfunction [5, 10].

Pulmonary valve morphology and stenosis severity were assessed using Doppler techniques, with pressure gradients calculated via the Bernoulli equation [11]. Tricuspid regurgitation velocity was utilised to estimate pulmonary artery systolic pressure, while LV systolic function was evaluated through standard two-dimensional methods [12].

### Three-dimensional echocardiography (3DE) protocol

After two-dimensional and Doppler assessment, electrocardiogram-gated 3DE was performed using a Vivid E95 system with a broadband X7-1 matrix-array transducer. Full-volume RV datasets were acquired from the apical four-chamber view using a four-beat acquisition and merged into a single dataset, ensuring complete RV inclusion with minimal artefacts. After optimising gain/zoom, alignment was adjusted so the long axis passed through the tricuspid valve centre and RV apex. Semi-automated RV quantification was then performed by placing six landmarks (two tricuspid annular points and the RV apex in the four-chamber view, and RV/LV anterior and posterior points plus an RV free-wall point in a mid short-axis view), followed by model review and manual contour correction when required, including RV outflow tract editing if needed [13]. Offline analysis identified end-diastolic and end-systolic frames, and RVEF was calculated as [(RVEDV − RVESV)/RVEDV] × 100, with RVEF ≥ 45% considered within the normal range [14].

### Outcomes

The primary outcome was RV systolic function at follow-up, assessed by 3D echocardiography as 3D RVEF, with RVEF < 45% defining impaired function. The main secondary outcomes were 3D RV volume indices (RVEDVi and RVESVi) and conventional 2D RV systolic indices (TAPSE, tissue Doppler S′, and FAC), as well as the residual pulmonary valve peak pressure gradient on Doppler.

### Statistical methods

Data management and statistical analysis were done using SPSS version 27 (IBM, Armonk, New York, United States). Quantitative data were assessed for normality using the Shapiro-Wilk test. According to normality, quantitative data were summarized as means and standard deviations or medians and ranges. Categorical data were summarised as numbers and percentages. Quantitative data were compared between the groups using the independent t-test and the Mann–Whitney U Test for parametric and non-parametric variables, respectively. Categorical data were compared using the Chi-square or Fisher’s exact test. All statistical tests were two-sided. P-values less than 0.05 were considered significant.

## Results

This study included 200 pediatric patients with isolated PVS who had undergone successful balloon pulmonary valvuloplasty at least 1 year prior to evaluation. For analysis, patients were divided based on 3D RV systolic function into group 1 (normal RV EF; 3D RVEF ≥ 45%, *n* = 142) and group 2 (impaired RV EF; 3D RVEF < 45%, *n* = 58).

Patients in group 2 (impaired 3D RV EF) were older than group 1 (normal 3D RV EF) (median 120 [60–204] vs. 72 [18–192] months, *P* < 0.001) and showed heavier body weight (34.21 ± 11.64 vs. 23.11 ± 8.27 kg, *P* < 0.001), height (138.17 ± 13.69 vs. 117.56 ± 20.64 cm, *P* < 0.001), and BSA (1.17 ± 0.26 vs. 0.87 ± 0.24 m², *P* < 0.001). They also underwent BPV at an older age (median 24 [8–168] vs. 3 [1.5–144] months, *P* < 0.001) and had a longer interval since intervention (median 96 [24–180] vs. 68 [16–190] months, *P* = 0.004). Table 1.

**Table 1:** Baseline demographic and anthropometric characteristics according to 3D RV EF status

		Total (*n* = 200)	RV EF		*P*-value
			Normal (*n* = 142)	Impaired (*n* = 58)	
**Age (months)**	Median (range)	102 (18–204)	72 (18–192)	120 (60–204)	< 0.001*
**Consanguinity**	n (%)	29 (29)	21 (29.6)	8 (27.6)	0.842
**Order of birth**	Median (range)	2 (1–6)	2 (1–6)	2 (1–6)	0.135
**Weigh (kg)**	Mean ± SD	26.33 ± 10.6	23.11 ± 8.27	34.21 ± 11.64	< 0.001*
**Height (cm)**	Mean ± SD	123.54 ± 21.03	117.56 ± 20.64	138.17 ± 13.69	< 0.001*
BSA (m²)	Mean ± SD	0.96 ± 0.28	0.87 ± 0.24	1.17 ± 0.26	< 0.001*
**Age of intervention (months)**	Median (range)	6 (1.5–168)	3 (1.5–144)	24 (8–168)	< 0.001*
**Time since intervention (months)**	Median (range)	84 (16–190)	68 (16–190)	96 (24–180)	0.004*

RV: right ventricle, EF: ejection fraction, RV EF: right ventricular ejection fraction, BSA: body surface area, SD: standard deviation, *: Significant P-value

Group 2 had higher pre-dilatation PPG than group 1 (97.24 ± 26.47 vs. 83.8 ± 24.61 mmHg, *P* = 0.017) and higher post-dilatation PPG (30.97 ± 9.81 vs. 23.56 ± 8.59 mmHg, *P* < 0.001). Balloon size was larger in group 2 (14.14 ± 4.07 vs. 12.2 ± 2.42, *P* = 0.004), and the balloon-to-annulus ratio was slightly higher (1.24 ± 0.15 vs. 1.22 ± 0.18, *P* = 0.047). Table 2.

**Table 2:** Balloon pulmonary valvuloplasty procedural characteristics and pulmonary valve peak pressure gradients according to 3D RV EF status

		Total (*n* = 200)	RV EF		*P*-value
			Normal (*n* = 142)	Impaired (*n* = 58)	
PPG (mmHg)					
Pre-dilatation	Mean ± SD	87.7 ± 25.77	83.8 ± 24.61	97.24 ± 26.47	0.017*
Post-dilatation	Mean ± SD	25.71 ± 5.72	23.56 ± 8.59	30.97 ± 9.81	< 0.001*
Size of balloon	Mean ± SD	12.76 ± 3.1	12.2 ± 2.42	14.14 ± 4.07	0.004*
Balloon annulus ratio	Mean ± SD	1.22 ± 0.18	1.22 ± 0.18	1.24 ± 0.15	0.047*

RV: right ventricle, EF: ejection fraction, RV EF: right ventricular ejection fraction, PPG: peak pressure gradient, *: Significant P-value

Group 2 had higher residual 2D post-procedure PPG (30.97 ± 9.81 vs. 23.56 ± 8.59 mmHg, *P* < 0.001) and lower 2D RV systolic indices: S′ (12.64 ± 3.22 vs. 15.01 ± 1.09 cm/s, *P* < 0.001), TAPSE (16.28 ± 2.75 vs. 19.21 ± 1.72 mm, *P* < 0.001), and FAC (39.86 ± 5.21 vs. 49.8 ± 2.62%, *P* < 0.001). Structurally, all patients in group 2 had RV dilatation (100% vs. 28.2%, *P* < 0.001) and greater RV wall thickness (9.19 ± 1.42 vs. 6.15 ± 1.20 mm, *P* < 0.001). LV EF was lower in the group 2 (63.34 ± 3.62 vs. 66.58 ± 4.72%, *P* = 0.001). On 3DE, group 2 had higher RV EDV index (62.0 ± 11.06 vs. 43.14 ± 11.44, *P* < 0.001), higher RV ESV index (34.41 ± 8.24 vs. 22.76 ± 6.65, *P* < 0.001), higher RV SV index (33.79 ± 7.25 vs. 24.76 ± 7.34, *P* < 0.001), and larger basal RV diastolic diameter (45.38 ± 11.31 vs. 39.2 ± 12.37, *P* = 0.022). 3D TAPSE (16.14 ± 3.01 vs. 19.76 ± 1.70, *P* < 0.001) and 3D RV FAC (39.64 ± 4.59 vs. 49.82 ± 2.70, *P* < 0.001) were also lower in group 2. Table 3.

**Table 3:** Two-dimensional and three-dimensional echocardiographic findings according to 3D RV EF status (row including PR severity deleted)

		RV EF			*P*-value
		Total (*n* = 200)	Normal (*n* = 142)	Impaired (*n* = 58)	
**2D PPG post**	Mean ± SD		23.56 ± 8.59	30.97 ± 9.81	< 0.001*
**2D RV S’**	Mean ± SD	14.33 ± 2.22	15.01 ± 1.09	12.64 ± 3.22	< 0.001*
**RV function acc. To 2D S’**					
Normal	n (%)	180 (90)	142 (100.0)	38 (65.5)	< 0.001*
Impaired	n (%)	20 (10)	0 (0.0)	20 (34.5)	
**Dimension**					
Normal	n (%)	102 (51)	102 (71.8)	0 (0.0)	< 0.001*
Dilated	n (%)	98 (49)	40 (28.2)	58 (100.0)	
**RV wall thickness**	Mean ± SD	7.04 ± 1.87	6.15 ± 1.2	9.19 ± 1.42	< 0.001*
**2D TAPSE**	Mean ± SD	18.36 ± 2.46	19.21 ± 1.72	16.28 ± 2.75	< 0.001*
**RV function acc. To 2D TAPSE**					
Normal	n (%)	180 (90)	142 (100.0)	38 (65.5)	< 0.001*
Impaired	n (%)	20 (10)	0 (0.0)	20 (34.5)	
**2D FAC %**	Mean ± SD	46.92 ± 5.75	49.8 ± 2.62	39.86 ± 5.21	< 0.001*
**RV function acc. To 2D FAC %**					
Normal	n (%)	180 (90)	142 (100.0)	38 (65.5)	< 0.001*
Impaired	n (%)	20 (10)	0 (0.0)	20 (34.5)	
**2D LV EF %**	Mean ± SD	65.64 ± 4.65	66.58 ± 4.72	63.34 ± 3.62	0.001*
**3D RV EDV Index**	Mean ± SD	48.61 ± 14.18	43.14 ± 11.44	62 ± 11.06	< 0.001*
**(f)64.3 ± 15.8 (m)70.5 ± 16.0**					
Normal	n (%)	180 (90)	140 (98.6)	40 (69.0)	< 0.001*
High Normal	n (%)	20 (10)	2 (1.4)	18 (31.0)	
**3D RV ESV Index**	Mean ± SD	26.14 ± 8.87	22.76 ± 6.65	34.41 ± 8.24	< 0.001*
**(f)27.7 ± 8.0 (m)30.4 ± 8.1**					
Normal	n (%)	174 (87)	140 (98.6)	34 (58.6)	< 0.001*
High Normal	n (%)	26 (13)	2 (1.4)	24 (41.4)	
**3D RV SV Index**	Mean ± SD	27 ± 8	24.76 ± 7.34	33.79 ± 7.25	< 0.001*
**(f)36.6 ± 9.0 (m)40.1 ± 9.2**					
Normal	n (%)	162 (81)	104 (73.2)	58 (100.0)	0.002*
Low	n (%)	38 (19)	38 (26.8)	0 (0.0)	
**3D RV Dd base**	Mean ± SD	40.99 ± 12.34	39.2 ± 12.37	45.38 ± 11.31	0.022*
**3d RV Dd mid**	Mean ± SD	35.23 ± 9.36	35.37 ± 10.43	34.9 ± 6.14	0.821
**3D RV Ld**	Mean ± SD	52.17 ± 12.16	51.21 ± 11.26	54.52 ± 14.06	0.219
**3D TAPSE**	Mean ± SD	18.71 ± 2.71	19.76 ± 1.7	16.14 ± 3.01	< 0.001*
**3D RV FAC**	Mean ± SD	46.86 ± 5.71	49.82 ± 2.7	39.64 ± 4.59	< 0.001*

RV: right ventricle, EF: ejection fraction, RV EF: right ventricular ejection fraction, 2D: two-dimensional, 3D: three-dimensional, PPG: peak pressure gradient, PR: pulmonary regurgitation, Mod: moderate, S′: tricuspid annular systolic velocity, TAPSE: tricuspid annular plane systolic excursion, FAC: fractional area change, LV: left ventricle, LV EF: left ventricular ejection fraction, EDV: end-diastolic volume, ESV: end-systolic volume, SV: stroke volume, Dd: diastolic diameter, Ld: longitudinal dimension, f: female, m: male, *: Significant P-value

2D echocardiography could detect RV dysfunction in only 10% of patients while 3D echo could detect RV EF impairment in 29% shown in Table 4.

**Table 4:** 2D Echo post dilatation of the studied patients

4) Echo post dilatation		Total no.=200
2D RV S’	Mean ± SD	14.33 ± 2.22
	Range	7.5–18
RV function acc. to S’	Normal	180 (90%)
	Impaired	20 (10%)
TAPSE	Mean ± SD	18.36 ± 2.46
	Range	10–23
RV functions acc. To TAPSE	Normal	180 (90%)
	Impaired	20 (10%)
FAC %	Mean ± SD	46.92 ± 5.75
	Range	30–56
RV functions acc. To FAC %	Normal	180 (90%)
	Impaired	20 (10%)

Positive significant statistical correlation was found between RV EF and 2D RV S’, TAPSE, FAC %, LV EF %, TAPSE and RV FAC by 3D echo. Also, there was a statistically significant negative correlation found between RV EF and RV EDV, RV ESV, RV SV, age (month), weigh (Kg), height (cm), BSA (m2), time since intervention (month), age of intervention (month), PPG, invasive baseline PG, size of balloon, balloon annulus ratio, number of inflations, pull back PR gradient post-dilatation, PG and RV wall thickness. Shown in Table 5.

**Table 5:** Correlation of 3D RV Volumes & functions with the other studied parameters

	RV EDV		RV ESV		RV SV		RV EF	
	*r*	*P*-value	*r*	*P*-value	*r*	*P*-value	*r*	*P*-value
RV EDV	--	--	0.950**	**0.000**	0.893**	**0.000**	-0.581**	**0.000**
RV ESV	0.950**	**0.000**	--	--	0.851**	**0.000**	-0.565**	**0.000**
RV SV	0.893**	**0.000**	0.851**	**0.000**	--	--	-0.469**	**0.000**
RV EF	-0.581**	**0.000**	-0.565**	**0.000**	-0.469**	**0.000**	--	--
Age(month)	0.950**	**0.000**	0.549**	**0.000**	0.531**	**0.000**	-0.502**	**0.000**
Order of birth	--	0.584	0.072	0.475	0.061	0.544	-0.113	0.263
Weight (Kg)	0.851**	**0.000**	0.501**	**0.000**	0.484**	**0.000**	-0.440**	**0.000**
Height(cm)	-0.565**	**0.000**	0.504**	**0.000**	0.524**	**0.000**	-0.461**	**0.000**
BSA (m2)	0.549**	**0.000**	0.490**	**0.000**	0.494**	**0.000**	-0.468**	**0.000**
Age of intervention (monthrespectively.)	0.226*	**0.024**	0.151	0.134	0.160	0.111	-0.428**	**0.000**
Time since intervention (month)	0.533**	**0.000**	0.539**	**0.000**	0.509**	**0.000**	-0.303**	**0.002**
PPG	0.145	0.149	0.211*	**0.035**	-0.001	0.994	-0.251*	**0.012**
Invasive PG predilatation	0.171	0.089	0.248*	**0.013**	0.029	0.773	-0.270**	**0.007**
Annulus	0.071	0.484	0.002	0.982	0.035	0.729	-0.188	0.062
Size of balloon	0.086	0.396	0.046	0.649	0.046	0.647	-0.311**	**0.002**
Balloon annulus ratio	0.010	0.921	0.102	0.311	0.011	0.915	-0.239*	**0.017**
Number of inflations	0.181	0.071	0.200*	**0.046**	0.066	0.511	-0.363**	**0.000**
Pull back pr gradient post dilatation	0.056	0.582	0.079	0.437	-0.034	0.739	-0.219*	**0.029**
PG	0.058	0.569	0.126	0.213	-0.044	0.666	-0.409**	**0.000**
2D RV S’	-0.139	0.167	-0.148	0.141	-0.044	0.663	0.542**	**0.000**
RV wall thickness	0.604**	**0.000**	0.541**	**0.000**	0.468**	**0.000**	-0.721**	**0.000**
TAPSE	-0.242*	**0.015**	-0.204*	**0.042**	-0.096	0.342	0.637**	**0.000**
FAC %	-0.523**	**0.000**	-0.504**	**0.000**	-0.416**	**0.000**	0.949**	**0.000**
LV EF %	-0.257**	**0.010**	-0.232*	**0.020**	-0.248*	**0.013**	0.238*	**0.017**
3D RV Dd base	0.373**	**0.000**	0.333**	**0.001**	0.393**	**0.000**	-0.155	0.122
3d RV Dd mid	0.288**	**0.004**	0.289**	**0.004**	0.334**	**0.001**	0.040	0.691
3D RV Ld	0.254*	**0.011**	0.235*	**0.018**	0.347**	**0.000**	-0.082	0.418
TAPSE	-0.285**	**0.004**	-0.262**	**0.008**	-0.133	0.187	0.685**	**0.000**
RV FAC	-0.568**	**0.000**	-0.549**	**0.000**	-0.456**	**0.000**	0.957**	**0.000**

P-value > 0.05: Non-significant (NS); P-value < 0.05: Significant (S); P-value < 0.01: highly significant (HS)

By furtherly studying the correlation of these variables with RV EF using multivariate linear regression analysis, the only factors that could show independent relation affecting the value of RV EF were found to be RV FAC %by 2D echo and FAC% by 3D echo as shown in Table 6.

**Table 6:** Multi-variate liner regression for factors affecting the value of RV EF

	Unstandardized Coefficients		Standardized Coefficients	t	*P*-value
	B	SE	Beta		
Age(month)	0.010	0.050	0.102	0.193	0.848
Weight (Kg)	-0.014	0.050	-0.033	-0.284	0.777
Height(cm)	0.004	0.027	0.021	0.167	0.867
BSA (m2)	1.369	2.698	0.084	0.507	0.613
Age of intervention (month)	-0.011	0.049	-0.070	-0.229	0.820
Time since intervention (month)	-0.014	0.047	-0.135	-0.305	0.761
PPG	0.007	0.034	0.041	0.211	0.833
Invasive PG pre dilatation	0.009	0.035	0.046	0.244	0.808
Size of balloon	-0.019	0.055	-0.013	-0.339	0.736
Balloon annulus ratio	0.892	1.389	0.025	0.642	0.523
Number of inflations	0.211	0.440	0.023	0.480	0.632
Pull back pr gradient post dilatation	0.016	0.037	0.020	0.434	0.666
PG	-0.037	0.029	-0.077	-1.272	0.207
2D RV S’	-0.208	0.109	-0.102	-1.910	0.060
RV wall thickness	-0.115	0.131	-0.047	-0.873	0.385
LV EF %	0.000	0.036	0.000	-0.004	0.997
RV EDV	0.001	0.027	0.003	0.024	0.981
RV ESV	-0.035	0.037	-0.112	-0.946	0.347
RV SV	0.030	0.035	0.063	0.847	0.399
TAPSE	-0.047	0.098	-0.028	-0.476	0.635
RV FAC	0.428	0.163	0.538	**2.627**	**0.010**

## Discussion

In this study, long-term RV systolic performance following successful BPV was heterogeneous. Patients classified as having impaired 3D-derived RVEF exhibited less favourable RV remodelling and lower conventional RV systolic indices, alongside higher residual pulmonary valve gradients. Differences in age at intervention and in the duration of follow-up were also observed between functional groups, suggesting that the timing of relief and cumulative exposure may shape late RV recovery.

Regarding late RV systolic function detected by advanced imaging, prior studies support the concept that abnormalities may persist despite an adequate early reduction in obstruction. In a late follow-up cohort of adolescents and young adults after balloon pulmonary valvuloplasty, Li and colleagues [15] reported reduced RV longitudinal systolic strain compared with controls, with additional evidence of LV mechanical involvement; their data suggest that measurable ventricular dysfunction can remain detectable years after intervention even when routine clinical follow-up appears reassuring. In another late follow-up study, de Azevedo et al. [16] evaluated patients after BPV and found that RV GLS was abnormal in 8 of 18 patients, whereas conventional methods were abnormal in only 2 of 18, illustrating the higher sensitivity of myocardial deformation for detecting residual systolic impairment in long-term surveillance. Together, these reports align with the broader observation that global or deformation-based measures often identify persistent RV dysfunction even when single-plane indices remain within reference ranges.

In terms of RV remodelling and chamber enlargement in late follow-up, CMR and echocardiographic literature consistently describe a subset of patients with sustained RV dilation. Harrild and colleagues used CMR late after balloon dilation and reported RV dilation (RV end-diastolic volume z-score ≥ 2) in 14 of 35 patients (40%). In their cohort, pulmonary regurgitation fraction correlated strongly with indexed RV end-diastolic volume, supporting a close link between regurgitant load and volumetric remodelling. In the study by Li et al. [15], patients had larger RV end-diastolic and end-systolic volumes than controls on advanced imaging, reinforcing that chamber enlargement may persist well beyond the early post-procedure period.

According to 3DE-derived RV volumes and ejection fraction in peri- and post-intervention studies, early remodelling after relief of pressure overload can be modest and parameter dependent. Mansour et al. [17] assessed children with congenital pulmonary stenosis by 3DE before BPV and at 3 months, reporting an increase in 3D RV ejection fraction from 50.72% ± 8.07% to 53.74% ± 9.57%. In the same study, indexed 3D RV end-diastolic volume increased from 28.77 ± 16.46 to 30.52 ± 15.03 ml/m², while indexed 3D RV end-systolic volume remained essentially unchanged (14.40 ± 8.86 to 14.45 ± 8.52 ml/m²). This pattern is consistent with an early phase in which afterload reduction may improve global systolic performance without immediate reversal of chamber geometry. When considered alongside longer-term CMR observations of RV dilation in some patients—particularly when PR is present—these data support a trajectory where early functional improvement does not preclude later volumetric remodelling.

Regarding residual stenosis, gradients, and re-intervention across long-term cohorts, large follow-up series show that a notable minority have persistent or recurrent obstruction. In a multicentre study with follow-up extending up to 25 years, Hansen et al. [18] reported residual pulmonary stenosis > 40 mmHg in 41 of 254 patients (17%) and re-intervention in 31 (13%). They also reported that higher initial pulmonary stenosis gradients were associated with long-term residual stenosis or repeat intervention, highlighting that baseline hemodynamic severity can shape long-term valve physiology even after catheter-based relief. Behjati-Ardakani et al. [19] reported durable gradient reduction in long-term follow-up, with pulmonary valve systolic pressure gradient decreasing from 83.3 ± 32.1 mmHg before BPV to 19.3 ± 14.2 mmHg immediately after the procedure and to 12.3 ± 6.6 mmHg at late follow-up. Collectively, the literature supports a spectrum in which many patients achieve sustained relief, but residual or recurrent gradients remain relevant in long-term surveillance and may coexist with functional variability.

Regarding conventional RV systolic indices (TAPSE and FAC) and their evolution after BPV, published data suggest that these measures may improve over time, but they do not always parallel advanced measures. In an acute post-BPV assessment using speckle tracking, Sirico et al. reported an early increase in FAC from 40.11% to 44.42% within 24 h, while TAPSE and RV FWS did not show the same immediate improvement, indicating that short-term changes may differ across components of RV contraction. In the 3-month follow-up study by Mansour et al. [17]Both FAC and TAPSE increased (FAC from 51.99% ± 9.58% to 55.41% ± 10.02%; TAPSE from 21.58 ± 3.22 to 22.50 ± 3.41 mm), supporting the view that conventional indices can show measurable improvement after the early phase. By contrast, de Azevedo et al. [16] demonstrated that conventional indices were abnormal in only 2 of 18 patients while strain abnormalities were present in 8 of 18, highlighting that routine parameters may underestimate late dysfunction in a subset of patients. Across studies, conventional indices remain useful, but advanced measures often detect residual impairment that conventional indices may miss [5, 14–16].

According to ventricular–ventricular interaction and LV consequences in late post-BPV cohorts, external studies suggest that long-term RV remodelling can be accompanied by measurable LV mechanical changes. Li et al. [15] reported lower LVEF in patients compared with controls and demonstrated associations between RV volumes and LV circumferential strain and dyssynchrony, implying that altered RV geometry and mechanics may influence LV performance through interventricular coupling. This supports the relevance of assessing both ventricles when interpreting long-term post-BPV physiology, particularly in patients with persistent RV dilation or functional impairment on advanced imaging.

Finally, this study had its limitations. First, its cross-sectional design cannot track RV recovery over time. Second, baseline/early post-BPV 3DE data were unavailable. Third, this was a single-centre cohort without CMR validation. Finally, RV measures may have been influenced by residual gradients and pulmonary regurgitation. Therefore, prospective multicenter studies with standardised, serial 3DE (and CMR validation when feasible) are recommended to define RV remodelling trajectories after BPV, clarify the impact of residual gradients and pulmonary regurgitation, and identify thresholds that should trigger closer follow-up or earlier intervention. There is no published literature studying the predictors of long-term RV 3D EF. Some studies considered the long-term effects of the procedure on restenosis rates and the prevalence of pulmonary regurgitation without accounting for RV function using 3D imaging, as in *Merino-*Ingelmo et al. (2014) [20].

]Other studies assessed and compared the RV 3D parameters at baseline and at short to intermediate-term follow-up (3 months), yet without considering the parameters influencing improvement as Mansour et al. (2020) [17].

Thus, in this sense, we see our work as pilot research that may impact future BPV indications and open the door to larger randomised controlled trials that could alter guideline recommendations.

## Conclusions

At mid to long-term follow-up after successful BPV for isolated PVS, the procedure is highly successful, with positive long-term outcomes. Several modifiable and non-modifiable factors could affect the long-term 3D-derived RV EF.

3DE identified impaired RV systolic function in nearly one-third of children. Lower RVEF was accompanied by RV dilatation and consistently lower conventional RV indices (S′, TAPSE, and FAC). It also showed a possible association with older age at intervention and higher residual gradients. However, independent relation couldn’t be established when applying multivariate linear regression analysis. Patients with RV impairment were underestimated when assessed by 2D vs. 3D echo, these findings support incorporating 3DE-derived RV volumes and RVEF into routine post-BPV surveillance to better detect persistent dysfunction and refine risk stratification.

### Recommendations

We suggest that balloon pulmonary valvuloplasty at an early age (< 3 months), before the development of higher systolic gradients across the pulmonary valve, using a lower balloon-to-annulus ratio could achieve the most favorable long-term outcomes.

### Limitations

- 3D echocardiography has several known limitations, mainly unfavourable anatomy and positioning of the right ventricle (RV), especially in cases of markedly dilated RV. In uncooperative pediatric patients who cannot hold their breath, the lack of single-beat 3D echocardiography during volume acquisition can lead to stitch artefacts. One potential solution is to reduce the number of beats while maintaining an appropriate frame rate.
- Additionally, the large 4 V probe (GE Vivid™ E95) may not fit between the intercostal spaces of small children. Developing a pediatric 3D transthoracic probe for single-beat acquisition could help address these technical challenges.
- The heterogenous age and body size distribution of the study population might influence the interpretation of echocardiographic measurements. Although RV parameters were reported as absolute values and where appropriate indexed to body surface area, age and body adjusted Z scores were not used. This may limit age related deviations from normal values, particularly for RV dimensions. Future studies incorporating appropriate pediatric reference models may provide additional insights into the interpretation of these measurements.(paragraph added).
- Currently, there are no established normal pediatric values for RV EF, FAC, and RV volumes. Future studies aimed at establishing these values in normal pediatric populations may prove beneficial, as reference values for pediatric echocardiography can differ significantly from those of adults.

## Data Availability

All data supporting the findings of this study are available within the paper and its Supplementary Information.
